# Transcriptome data of oil palm dumpy seedlings treated with different concentration of phosphorus in hydroponic system

**DOI:** 10.1016/j.dib.2026.112968

**Published:** 2026-06-15

**Authors:** Sigit Dwi Maryanto, Zulfikar Achmad Tanjung, Roberdi Roberdi, Tri Rini Nuringtyas, Purnomo Purnomo, Diah Rachmawati, Eka Tarwaca Susila Putra, Condro Utomo, Tony Liwang, Budi Setiadi Daryono

**Affiliations:** a Doctoral Program, Department of Biology, Faculty of Biology, Universitas Gadjah Mada. Jl. Teknika Selatan, Sekip Utara, Sleman, Yogyakarta 55281, Indonesia; b Biotechnology Department, Plant Production and Biotechnology Division, PT SMART Tbk. Jl. Raya Cijayanti, Babakan Madang, Bogor 16810, West Java, Indonesia; c Faculty of Biology, Universitas Gadjah Mada. Jl. Teknika Selatan, Sekip Utara, Sleman, Yogyakarta 55281, Indonesia; d Faculty of Agriculture, Universitas Gadjah Mada. Jl. Flora, Bulaksumur, Yogyakarta 55281, Indonesia

**Keywords:** Hydroponic, Oil palm, P-dosage, RNAseq, Transcriptome data

## Abstract

Phosphorus (P) is the second most utilised macronutrient in oil palm plantations. Employing a molecular genetic approach to develop oil palm genotype with improved P uptake efficiency could mitigate phosphorus deficiency. The molecular mechanisms underlying the roles of Phosphorus in oil palm remain are not fully understood. A comprehensive study of gene regulation patterns concerning phosphorus dosages will be useful for elucidating the biological processes and cellular mechanisms in oil palm. In this study, RNA sequencing (RNAseq) analysis was performed using Dumpy (DyP)-progeny treated with five different P dosages. Three-months-old Dumpy seedlings were grown hydroponically and maintained under five P dosages for 180 days. P concentration treatments including P starvation (P0: 0% (v/v)), P deficiency (P30: 1.40%; P65: 3.03%; P100: 4.67% (v/v)) and P optimum (P300: 14.02% (v/v)). P300 (14.02%) is an optimum P dosage for oil palm seedlings in the main nursery stage based on an internal standard procedure. The leaf samples were collected at the end of the trial and the RNA was isolated and subjected to RNA sequencing. The Illumina NextSeq 2000 platform was applied to generate a total nucleotide sequence of approximately 23.2 Gb. A total of 84,285,330 raw reads were obtained and submitted to the NCBI Sequence Read Archive (SRA) repository under the BioProject accession number PRJNA1217601. This dataset will be a valuable resource for identifying genes associated with different P supplies. Moreover, the transcriptome data obtained will be valuable for the development of potential SNP molecular markers for P uptake efficiency in oil palm.

Specification Table

**Table:** 

Subject	Biology; Plant physiology; Nutrient
Specific subject area	*RNAseq data of leaf of oil palm seedlings treated with five different P dosages including P0: 0% (v/v) represent starvation; P30: 1.40%, P65: 3.03%, P100: 4,67% (v/v) represent deficiency and P300: 14.02% (v/v) represent optimum dosage) in hydroponic system. An optimum P dosage was based on internal procedure for oil palm seedling in main nursery stage.*
Type of data	*Raw reads in FASTQ format*.
Data collection	*Leaf samples of oil palm seedlings grown in a hydroponic system were collected 180 days after treatment (DAT). The seedlings were treated with five dosages of P. Trial was conducted in a greenhouse at the Faculty of Biology, Gadjah Mada University, Special Region of Yogyakarta, Indonesia. Leaf RNA was isolated using RNAeasy Plant Miny Kit (Qiagen, Hilden, Germany). mRNA was enriched from total RNA using a poly A selection module. The enriched mRNA was fragmented using enzymatic methods to match the expected insert sizes. The fragmented DNA was ligated to an Illumina-compatible adapter. PCR was conducted to amplify the library and generate the full-length adaptor containing a unique index sequence in each library sample. The quality and quantity of the library samples were determined using Tape station and qPCR. The library samples were sequenced with 300 cycles (150 PE). The raw reads were generated using the Illumina NextSeq 2000 platform. Demultiplexing and Fastq generation was performed using Illumina DRAGEN BCL Convert-4.2.7 of NextSeq 2000 instrument. FASTP and Kallisto software with default parameters were used for data analysis.*
Data source location	*City: Yogyakarta; Country: Indonesia*
Data accessibility	Repository name: Sequence Read Archive (SRA), NCBI Data identification number: PRJNA1217601 Direct URL to data: https://www.ncbi.nlm.nih.gov/bioproject/?term=PRJNA1217601 The original data was deposited in NCBI’s repository under BioProject accession number PRJNA1217601 (NCBI)
Related research article	*none.*

## 1 Value of Data

• The complete sets of transcriptome profiles of the leaves of oil palm seedlings treated with five dosages of P, namely P0: 0% (v/v); P30: 1.40% (v/v); P65: 3.03% (v/v); P100: 4.67% (v/v) and P300: 14.02% (v/v) in hydroponic system for 180 days were obtained.
• This information is valuable for understanding the cellular and molecular mechanisms underlying phosphate uptake in oil palm seedlings.
• The transcriptome data profiles combined with oil palm genome sequences will be used to identify functional molecular markers such as single nucleotide polymorphisms (SNP) and microsatellites that can be used for the future oil palm genetic improvement.

## 2 Background

Dumpy is one of commercial oil palm varieties in Indonesia developed from a cross of Deli Dura x Pisifera SP540T [1]. Dumpy is known to have a slow stem height increment and high bunch weight. In addition, to achieve a higher yield, oil palm requires optimum mineral nutrition supply, including P. P is an essential nutrient for oil palm growth [2] and important for oil palm root growth during the establishment and early growth stages [3]. A large amount of P is essential for maintaining photosynthetic carbon fixation, which directly affects the productivity of oil palm [4]. Moreover, P plays an important role in seedling growths, fresh fruit bunch (FFB) productions, oil contents, bunch weights, and oil palm defence mechanisms against biotic and abiotic stresses [5]. The symptoms of P deficiency in oil palms are stunted palms with short fronds, the palm trunk a pronounced pyramid shape with a reduce number of flower and bunches [6].

In contrast, Indonesian oil palm plantations are mostly planted in ultisol soil type that is most severely influenced by P deficiency because of its acidic nature and low P availability [7]. However, the molecular regulation of P deficiency in oil palm is not fully understood. One approach that can be applied to overcome this challenge is transcriptome profiling using RNA sequencing [8]. The aim of this study was to obtain transcriptome profiles of the Dumpy variety treated with different P dosages. This research is expected to accelerate the development of superior palm variety with slow stem increments and efficient P absorptions.

## 3 Data Description

The Dumpy (DyP) is one of the commercial oil palm progenies widely planted in Indonesian [1]. This transcriptome dataset comprises RNA-seq raw reads of leaves of three-months-old Dumpy seedlings grown hydroponically and maintained under five P dosages for 180 days, namely starvation (P0: 0% (v/v)), deficient (P30: 1.40%; P65: 3.03%; P100: 4.67% (v/v)) and optimum (P300: 14.02% (v/v)) conditions. The raw data were generated using the Illumina NextSeq 2000 platform. EG5 transcript was used as a reference genome [9]. The raw reads were deposited in SRA under BioProject accession number PRJNA1217601. The accession numbers for each P-dosage treatment are listed in Table 1. Table 2 summarise the sequencing and mapping results of each P-dosage variance. Results of mapped reads shows varied from 76.47% to 84.08% (Table 2). The highest percentage of transcriptome dataset oil palm Dumpy seedlings was obtained from the P300 (14.02%) treatment (Table 1).

**Table 2:** RNA-seq data sets of oil palm leaves of Dumpy progeny.

Progeny	P-dosages	Raw reads (paired-end)	Raw nucleotides (bp)	Clean reads (paired-end)	Clean nucleotides (bp)	High-quality reads (paired-end)	Percentage of high-quality reads (%)	Percentages of mapped (%)
D x P, Tenera, Dumpy (DyP)	P0 (0%) (Starvation)	16,709,855	4600,108,732	16,544,062	4543,689,179	16,544,062	99,01	81,64
	P30 (1.40%) (Deficiency)	16,718,461	4599,918,591	16,595,046	4545,635,233	16,595,046	99,26	83,08
	P65 (3.03%) (Deficiency)	17,114,565	4600,071,083	16,998,701	4541,268,520	16,998,701	99,32	81,79
	P100 (4.67%) (Deficiency)	17,339,678	4700,048,783	17,223,230	4641,955,615	17,223,230	99,33	76,47
	P300 (14.02%) (Optimum)	17,211,484	4700,006,818	16,924,291	4634,318,636	16,924,291	98,33	84,08
Total		**85,094,043**	**23,200,154,007**	**84,285,330**	**22,906,867,183**	**84,285,330**

**Table 1:** Description of transcriptome dataset oil palm Dumpy seedlings of five samples treated with different Phosphorus (P) dosage and submitted to the Sequence Read Archive (SRA).

Progeny	BioSample	P-dosages	Fastq ID
D x P, Tenera, Dumpy (DyP)	SAMN46495462	P0: 0% (v/v) (Starvation)	Dump-0_R1.fastq.gz
			Dump-0_R2.fastq.gz
	SAMN46495463	P30: 1.40% (v/v) (Deficiency)	Dump-30_R1.fastq.gz
			Dump-30_R2.fastq.gz
	SAMN46495464	P65: 3.03% (v/v) (Deficiency)	Dump-65_R1.fastq.gz
			Dump-65_R2.fastq.gz
	SAMN46495465	P100: 4.67% (v/v) (Deficiency)	Dump-100_R1.fastq.gz
			Dump-100_R2.fastq.gz
	SAMN46495466	P300: 14.02% (v/v) (Optimum)	Dump-300_R1.fastq.gz
			Dump-300_R2.fastq.gz

## 4 Experimental Design, Materials, and Methods

### 4.1 Plant materials

Three-month-old of Tenera (DxP) seedlings of the Dumpy variety treated with P0: 0% (starvation-P), P30: 1.40% (deficiency-P), P65: 3.03% (deficiency-P), P100: 4.67% (deficiency-P), and P300: 14.02% (optimum-P). The P dosages were applied based on the previous trial. P300 (14.02%) is a optimum dosage based on an internal standard procedure for oil palm in main nursery stage. Seedlings were grown in a hydroponic system in the greenhouse of the Faculty of Biology, Gadjah Mada University, Special Region of Yogyakarta, Indonesia.

### 4.2 Hydroponic trials

Seedlings exhibiting similar plant heights were selected and planted in a greenhouse with a hydroponic system. The greenhouse temperature was controlled at 28 – 30 °C. The nutrient solution was prepared following the Hoagland method with several modifications [10]. The P treatments were applied for 180 days. Leaf samples for RNA analysis were collected at 180 days after treatment (DAT) and immediately placed in RNA Later to preserve the RNA (Thermo Scientific, Massachusetts, USA).

### 4.3 RNA extraction, cDNA library preparation and transcriptome sequencing

Total RNA was isolated using a commercial RNeasy Plant Mini Kit (QIAgen, Hilden, Germany) according to manufacturer’s instructions. The extracted RNA was quantified using a NanoDropTM 2000c spectrophotometer (Thermo Scientific, Massachusetts, USA). RNA that fulfilled the requirement for sequencing was preserved using RNAstable (Biomatrica, California, USA) during shipment.

RNA was quantified using the 2100 Bioanalyzer Instrument (Agilent Company, USA) to calculate RNA integrity number, rRNA ratio, and RNA concentration. Furthermore, mRNA was isolated from total RNA using magnetic beads with Oligo (dT). mRNA was enriched from total RNA using a pol A selection module. The enriched mRNA was fragmented using enzymatic methods to match the expected insert sizes. The fragmented DNA was ligated to an Illumina-compatible adapter. PCR was conducted to amplify the library and generate the full-length adaptor containing a unique index sequence in each library samples. The quality and quantity of the library samples were determined using Tape station and PCR. The library samples were sequenced with 300 cycles (150 PE) using Illumina NextSeq 2000. Demultiplexing and Fastq generation were performed using Illumina DRAGEN BCL Convert-4.2.7 of NextSeq 2000 instrumnet [6].

### 4.4 Data analysis

FASTP with default parameters was applied for filtering and quality control of the sequence including phred quality: 15; unqualified bases: 40; Ns allowed: 5; length: 15; polyG tail trimming: enable; and adapter trimming: auto [11]. EG5 transcript was used as the oil palm reference transcriptomes [9]. Kallisto software was used to quantify transcript abundances using default parameter including bootstrap-samples: 0 and seed: 42 [12].

## Limitation

none.

## Ethics Statements

All authors declare that plants collection were in accordance with all relevant guidelines.

## Credit Author

**Sigit Dwi Maryanto**: conceived, designed, performed the experiments, wrote of original the paper draft, statistic and bioinformatic analysed the data; **Zulfikar Achmad Tanjung:** methodology, writing-review, statistic and bioinformatic analysed the data; **Roberdi:** methodology, writing-review, editing, and supervision; **Tri Rini Nuringtyas:** supervision; **Purnomo**: supervision; **Diah Rachmawati**: supervision; **Eka Tarwaca Susila Putra**: supervision; **Condro Utomo**: supervision; **Tony Liwang**: supervision and resources; **Budi Setiadi Daryono**: supervision and resources.

## Data Availability

NCBIBIOPROJECT:PRJNA1217601 (Original data).
